# Prevalence and magnitude of acidosis sequelae to rice-based feeding regimen followed in Tamil Nadu, India

**DOI:** 10.14202/vetworld.2018.464-468

**Published:** 2018-04-11

**Authors:** Rathinam Murugeswari, Chinnamani Valli, Raman Karunakaran, Venkatasubramanian Leela, Amaresan Serma Saravana Pandian

**Affiliations:** 1 Institute of Animal Nutrition, Kancheepuram, Tamil Nadu Veterinary and Animal Sciences University, Tamil Nadu, India; 2 Department of Animal Nutrition, Madras Veterinary College, Tamil Nadu Veterinary and Animal Sciences University, Chennai, Tamil Nadu, India; 3 Department of Veterinary Physiology, Madras Veterinary College, Tamil Nadu Veterinary and Animal Sciences University, Chennai, Tamil Nadu, India; 4 Department of Animal Husbandry Economics, Madras Veterinary College, Tamil Nadu Veterinary and Animal Sciences University, Chennai, Tamil Nadu, India

**Keywords:** acidosis, cooked rice, dairy cows, digestive disturbance, survey

## Abstract

**Background and Aim:**

In Tamil Nadu, a southern state of India, rice is readily available at a low cost, hence, is cooked (cooking akin to human consumption) and fed irrationally to cross-bred dairy cattle with poor productivity. Hence, a study was carried out with the objective to examine the prevalence of acidosis sequelae to rice-based feeding regimen and assess its magnitude.

**Materials and Methods:**

A survey was conducted in all the 32 districts of Tamil Nadu, by randomly selecting two blocks per districts and from each block five villages were randomly selected. From each of the selected village, 10 dairy farmers belonging to the unorganized sector, owning one or two cross-bred dairy cows in early and mid-lactation were randomly selected so that a sample size of 100 farmers per district was maintained. The feeding regimen, milk yield was recorded, and occurrence of acidosis and incidence of laminitis were ascertained by the veterinarian with the confirmative test to determine the impact of feeding cooked rice to cows.

**Results:**

It is observed that 71.5% of farmers in unorganized sector feed cooked rice to their cattle. The incidence of acidosis progressively increased significantly (p<0.05) from 29.00% in cows fed with 0.5 kg of cooked rice to 69.23% in cows fed with more than 2.5 kg of cooked rice. However, the incidence of acidosis remained significantly (p<0.05) as low as 9.9% in cows fed feeding regimen without cooked rice which is suggestive of a correlation between excessive feeding cooked rice and onset of acidosis. Further, the noticeable difference in the incidence of acidosis observed between feeding cooked rice and those fed without rice and limited intake of oil cake indicates that there is a mismatch between energy and protein supply to these cattle. Among cooked rice-based diet, the incidence of laminitis increased progressively (p<0.05) from 9.2% to 37.9% with the increase in the quantum of cooked rice in the diet.

**Conclusion:**

The study points out the importance of protein supplementation in rice-based feeding regimen to set right the mismatched supply between nitrogen and fermentable organic matter in the rumen. This research has practical implications for animal health, welfare, nutrition, and management

## Introduction

Dairying is one of the most ancient occupations of rural India contributing significantly to employment opportunities and supplemental income for small and marginal farmers and landless laborers [1]. Since time in memorial dairy farmers of Tamil Nadu, India, belonging to the unorganized sector have been feeding their native cattle breeds having low productive potential with available feed resources, namely, grains of pearl millet, sorghum, maize, broken rice, and agricultural by-products such as rice bran, oil cakes, stovers, and straw [2]. Most of the farmers in the semi-intensive rearing of dairy animals feed crop residues as a major source of roughage with one or two concentrate ingredients, while only a few farmers feed commercial concentrate mixtures to their animals [3].

In a survey to determine traditional feeding practices for cross-bred dairy cattle reared in Tamil Nadu, it was found that the animals were being fed on gruel based unbalanced feed [4], the gruel includes kitchen waste, table waste, etc., apart from the decanted gruel on cooking rice.

With the advent of cross-breeding with superior germplasm, many farmers continue the traditional feeding pattern along with supplemental oil cakes. However, the ever-rising cost of oil cakes has resulted in farmers feeding more of rice grains/rice bran with or without gruel causing lower protein leading to a mismatch between energy and nitrogen supply in the rumen [5]. Excess feeding of cereal grains or cereal flours or boiled cereal flour leads to acidosis with the symptoms of ruminal bloat, loose dung with a foul smell and undigested grains, laminitis and reproductive problem [6]. In Tamil Nadu, rice is readily available at a low cost, hence, and is cooked (cooking akin to human consumption) and fed to cross-bred dairy cattle. Although the impact of feeding cooked rice on the incidence of the metabolic disorder is well known, the extent of incidence of acidosis and nutritional interventions required to combat it is not established.

Hence, a study was carried out with the objective to examine the prevalence of acidosis sequelae to rice-based feeding regimen and assess its magnitude.

## Materials and Methods

### Ethical approval

This study does not require the approval of the Institute Animal Ethics Committee. However, samples were collected as per standard sample collection procedure without harm to the animals.

Informed consent has been obtained from all participants.

### Study area and sampling procedure

The study was carried across the season, throughout Tamil Nadu, India, with a cross-sectional survey of 3200 dairy farmers to document the feeding regimen, milk production and the incidence of acidosis in cross-bred dairy cattle. Tamil Nadu state consists of 32 districts and from each district, two blocks were randomly selected and from each block, and five villages were randomly selected [7]. From each of the selected village, 10 dairy farmers belonging to the unorganized sector, owning one or two crossbred dairy cattle, were selected through a stratified random sampling technique so that a sample size of 100 farmers per district was maintained. Cross-bred dairy cattle in milking stage from 1 to 7 months from these farmers formed the study material. All the animals were apparently free-form parasitic infestation, reared in a tropical environment at the backyard of farmer’s residence and fed with palatable good quality feed ingredients free from exogenous contamination.

### Data collection

Randomly selected farmers feeding traditional feed which includes with or without cooked rice to their dairy cattle were individually contacted by the veterinarian to elicit information on the feeding regimen, milk yield and the occurrence of acidosis through a pre-tested structured questionnaire.

While the feeding regimen and milk yield data were recorded as per the farmer’s declaration, the prevalence of acidosis was identified by the veterinarian by examining the animal for typical symptoms such as feces consistency and presence of undigested fiber/grain and gas bubbles and Laminitis [8]. Confirmation test was carried out in portable pH meter on rumen fluid immediately after collection through stomach tubes and pH falling <5.5 were considered as acidotic [9].

### Statistical analysis

The data were subjected to descriptive statistics and frequency analysis [10]. The one-way ANOVA of CRD was used for comparison of acidosis effect between groups through SPS software. p=0.05 was considered statistically significant, and 0.01 was considered as highly significant.

## Results

The survey report based on 3200 farmers, holding 5332 crosses bred dairy cattle are presented in Table-1. The dairy farmers who fed cooked rice to their cattle constituted 71.5%. This study revealed that the farm holding ranged from 1 to 2 cross-bred dairy cattle with milk yield varied highly significantly (p<0.01) and ranged from 3.5 to 8.8 L/day/cow. The farm holding and the milk yield increased with the plane of nutrition as reflected by the feeding regimen. However, 2286 of 5332 cows produced milk <5 L per day. The survey also revealed that among cooked rice based feeding regimen, feeding cooked rice along with rice bran, rice straw and allowing the cattle to graze was followed by the majority (19%) of the farmers who held 1218 cross-bred dairy cows which amounted to 22.84% of cows of this study.

**Table-1:** Feeding regimen, farm holding, and milk yield of crossbred dairy cattle maintained by unorganized dairy farmers of Tamil Nadu, India.

Feeding Regime	Number of Farmers assessed	Percentage of farmers adopting feeding regimen	Average number of cattle per farmer	Average milk yield (L/day/cow)*
Grazing and cooked rice	302	9.4	1	3.5
Grazing, green fodder, and cooked rice	372	11.6	1	4.2
Grazing, rice straw, and cooked rice	394	12.3	1	4.4
Grazing, rice straw, rice bran, and cooked rice	609	19.0	2	4.9
Rice straw, green fodder oil cake, and cooked rice	311	9.7	2	6.9
Rice straw, green fodder oil cake, rice bran, and cooked rice	176	5.5	2	7.3
Rice straw, grazing, rice bran, cooked rice, and concentrate mixture	124	3.9	2	8.8
Feeding regimen without cooked rice	912	28.5	2	6.7

* p<0.01 highly significant among all feeding regimen except in the feeding regimen 5 and 8, Total number of farms–3200, Total number of animals-5332

It was observed that the quantum of oil cake/concentrate fed to cattle remained constant at 500 g per cow per day while the quantum of cooked rice fed varied.

The quantum of cooked rice fed and incidence of acidosis in the animals under study are presented in Table-2. The results indicate that the overall incidence of acidosis in cross-bred dairy cattle fed with cooked rice was 37.76%. Majority of cross-bred dairy cows (2768) were fed with 0.5-1 kg of cooked rice, and it constituted 51.91% of the surveyed cross-bred dairy cattle. The incidence of acidosis progressively increased significantly (p<0.05) from 29.00% in cows fed with 0.5 kg of cooked rice to 69.23% in cows fed with more than 2.5 kg of cooked rice. However, the incidence of acidosis remained significantly (p<0.05) as low as 9.9% in cows not fed with cooked rice which is suggestive of a correlation between excessive feeding cooked rice and onset of acidosis. It is also inferred that the magnitude of acidosis increases with corresponding increase in the quantity of cooked rice fed to crossbred dairy cattle.

**Table-2:** Quantity of rice (cooked) fed and incidence of acidosis in the study animals

Sl. No	Quantity of Cooked rice fed (Kg/Cow/day)	No of crossbred dairy cows	Percentage of crossbred dairy cows	Average pH of rumen fluid	No of crossbred dairy cows affected with acidosis	Percentage of crossbred dairy cows affected with acidosis*	Percentage of crossbred dairy cows affected with laminitis*
1	0.0	1728	32.41	6.5	171	9.90	2.9
2	0.5	1538	28.84	6.2	446	29.00	9.2
3	1.0	1230	23.07	6.0	476	38.70	11.8
4	1.5	508	9.53	6.0	249	49.02	15.7
5	2.0	243	4.56	5.9	136	55.97	27.8
6	2.5	59	1.11	5.7	36	61.02	29.4
7	Above 2.5	26	0.49	5.7	18	69.23	37.9

* p<0.05 among treatment group

The incidence of laminitis in cows was 2.9% in the diet not fed with cooked rice and differed significantly (p<0.05) with cooked rice-based diet. Among cooked rice-based diet, the incidence of laminitis increased progressively (p<0.05) from 9.2% to 37.9% with the increase in the quantum of cooked rice in the diet.

## Discussion

In Tamil Nadu, most dairy farms belong to the unorganized sector [11], and consequently, they utilize locally available feed resources for feeding their cows. Attempts to improve the feeding practices have met with little success due to the widespread nature of the problem and adoption of region-oriented traditional feeding practices [12]. Further farmers share their staple food (cooked rice) with cows due to special religious sentiment, besides in Tamil Nadu, rice is available at a very subsidized cost to economically weaker sections, which also largely depend on cows for their survival. Hence, 71.5% of the unorganized dairy farmers feed cooked rice to their cattle. Cooked rice is fed alone or along with other feed ingredients, namely, other cereals, oil cakes, and rice bran [13] which is amply reflected in this survey.

The results of this survey (42.87% of dairy cattle produce <5 l of milk) correlate with the observation recorded [14] in the unorganized farms, which indicated that majority of cattle are reared with unbalanced feeding regimen and consequently yield low milk lower than their genetic potential.

The overall incidence of acidosis (37.76%) in cross-bred dairy cattle fed with cooked rice recorded in this study concurred with the observations made else-where [15-17]. The incidence of ruminal acidosis due to the intake of a large amount of cooked rice and rice gruel has been reported in the neighboring country as well [18]. Rumen acidosis is associated with the feeding of diets with higher amounts of grain [19]. Feeding feeds rich in starch is the cause of ruminal acidosis in ruminants [20]. A very wet diet with highly fermented feeds is also prone to induce acidosis [21]. Similarly, the excessive amount of nonstructural carbohydrates with low neutral detergent fiber ingestion leads to acidosis [22]. Consumption of a large amount of rapidly fermentable carbohydrates, primarily starches, and sugars lead to ruminal acidosis [23]. In Bangladesh, it is reported that among the risk factors of ruminal acidosis, feeding of cooked rice was highest (50% and 62.5% in cattle and goats, respectively) followed by feeding of rice gruel (25%) and feeding jackfruit residue, potato, and bread each by 12.5% [24].

Cooked rice has rapidly fermentable starch and less protein which leads to the production of short-chain volatile fatty acids and lactic acid in the rumen [25]. The available carbohydrate in rice is reported to be 71.1% [26] whereas it is recommended not to exceed 36% in a feed containing rapidly digestible starch. It is advocated to provide adequate degradable protein to ferment available carbohydrate in the ration to increase microbial protein production and reduces the fermentation of energy to acids [27] and thereby reducing the risk of acidosis [28]. Excess feeding of cereal grains or cereal flours or boiled cereal flour leads to Subacute Ruminal Acidosis, which requires the synchronized release of nitrogen to divert carbon and hydrogen present in the fermentable organic matter to synthesize microbial protein [29]. Ruminal acidosis is defined as a fermentation disorder in the rumen characterized by a lower than normal ruminal pH, reflecting an imbalance between microbial production, microbial utilization, and ruminal absorption of volatile fatty acids [30].

Further, the noticeable difference in the incidence of acidosis observed between feeding cooked rice and those not fed with cooked rice and limited intake of oil cake indicates that there is a mismatch between energy and protein supply to these cattle apart from undernourishment of nutrients [31]. This indicates that a source of nitrogen needs to be supplemented for energy protein synchrony to augment microbial biomass production [19]. A major energy source of organic matter is carbohydrate and nitrogen can be non-protein nitrogen sources or protein sources from oil cakes for microbial protein synthesis [32]. Acidosis can be prevented by feeding management such as reducing the high grain diet and feeding adequate quantity of crude protein with energy for effective utilization of nutrients in the rumen [22].

The incidence rate of laminitis from surveys concurs with the reports that ranged from 5.5% to 30% [33]. Nutritional management has been identified as a key component in the development of laminitis, particularly the feeding of diets high in fermentable carbohydrates, which can result in an acidotic state. The lactic acid and the endotoxins and histamine released as the rumen flora die are absorbed systemically and affect the microvasculature of the growing hoof wall, which can then result in clinical laminitis [34].

## Conclusion

The survey report indicates that more than two-thirds of farmers in unorganized sector feed cooked rice and majority of them feed 0.5-1 kg per day per cow in Tamil Nadu. The incidence of acidosis (37.76%) and consequently laminitis ranging from 9.2% to 37.9% are widely prevalent in cooked rice-based feeding regimen and are more pronounced (p<0.05) as the quantity of cooked rice increase. Cooked rice-based feeding regimen with low quantity and quality of crude protein as practiced by farmer leads to a mismatch of energy and protein and consequently acidosis in dairy cattle. The study points out the importance of protein supplementation in rice-based feeding regimen to set right the mismatched supply between nitrogen and fermentable organic matter in the rumen.

## Authors’ Contributions

All the authors conceptualized the manuscript. Both RM and CV drafted the manuscript. RK and VL have given the intellectual suggestions. ASSP has done the statistical analysis part. RM and CV critically reviewed the manuscript. All the authors have read and approved the final version of the manuscript.
